# A Flow Cytometry-Based Assay for Quantifying Non-Plaque Forming Strains of Yellow Fever Virus

**DOI:** 10.1371/journal.pone.0041707

**Published:** 2012-09-19

**Authors:** Erika Hammarlund, Ian J. Amanna, Melissa E. Dubois, Alex Barron, Flora Engelmann, Ilhem Messaoudi, Mark K. Slifka

**Affiliations:** 1 Oregon National Primate Research Center, Oregon Health and Science University, Beaverton, Oregon, United States of America; 2 Najít Technologies, Inc., Beaverton, Oregon, United States of America; 3 Vaccine and Gene Therapy Institute, Oregon Health and Science University, Beaverton, Oregon, United States of America; University of Montreal, Canada

## Abstract

Primary clinical isolates of yellow fever virus can be difficult to quantitate by standard *in vitro* methods because they may not form discernable plaques or induce a measurable cytopathic effect (CPE) on cell monolayers. In our hands, the Dakar strain of yellow fever virus (YFV-Dakar) could not be measured by plaque assay (PA), focus-forming assay (FFA), or by measurement of CPE. For these reasons, we developed a YFV-specific monoclonal antibody (3A8.B6) and used it to optimize a highly sensitive flow cytometry-based tissue culture limiting dilution assay (TC-LDA) to measure levels of infectious virus. The TC-LDA was performed by incubating serial dilutions of virus in replicate wells of C6/36 cells and stained intracellularly for virus with MAb 3A8.B6. Using this approach, we could reproducibly quantitate YFV-Dakar in tissue culture supernatants as well as from the serum of viremic rhesus macaques experimentally infected with YFV-Dakar. Moreover, the TC-LDA approach was >10-fold more sensitive than standard plaque assay for quantitating typical plaque-forming strains of YFV including YFV-17D and YFV-FNV (French neurotropic vaccine). Together, these results indicate that the TC-LDA technique is effective for quantitating both plaque-forming and non-plaque-forming strains of yellow fever virus, and this methodology may be readily adapted for the study and quantitation of other non-plaque-forming viruses.

## Introduction

Despite the development of an effective vaccine in the late 1930's, yellow fever continues to represent an important emerging/re-emerging mosquito-borne disease responsible for approximately 200,000 infections and 30,000 deaths each year [1]. Endemic yellow fever is restricted mainly to countries in Africa and South America, but global travel may result in spread of the virus to other continents including North America. Indeed, the first recorded yellow fever outbreaks in British North America occurred in Boston, MA, Charleston, SC, and Philadelphia, PA in 1693 [2]. Moreover, yellow fever outbreaks continued to occur in the US throughout the 18^th^ and 19^th^ centuries and resulted in at least 19 major outbreaks in New York city alone [2]. In 1878, for example, a large yellow fever epidemic swept from the Gulf of Mexico up the Mississippi to Memphis and St. Louis, resulting in approximately 16,000–20,000 deaths [2]. In addition to North America, *Aedes* mosquito vectors competent for the spread of yellow fever can be found in parts of Asia, Australia, and Europe, theoretically putting a broader population at risk if the virus were to be inadvertently introduced into these regions [3], [4].

Although several vaccine strains of yellow fever (e.g., 17D, 17DD, FNV) can be readily quantitated by standard plaque assay methodologies [5], low passage clinical isolates of yellow fever may be more difficult to measure if they do not elicit plaque formation or induce a measurable cytopathic effect (CPE). Similar challenges have been faced with other flaviviruses such as dengue virus, in which clinical isolates often fail to induce CPE or form plaques [6]. To overcome these obstacles, several approaches to yellow fever virus quantitation have been developed including mouse intracranial LD_50_ (MICLD_50_) [7], fluorescence microscopy [8], focus forming assay [9], and quantitative real time PCR (qRT-PCR) [7], [10]–[12]. Here, we have developed a flow cytometry-based tissue culture limiting dilution assay for measuring infectious yellow fever virus and demonstrate that it works efficiently for measuring live virus from tissue culture as well as from viremic serum samples.

## Materials and Methods

### Ethics statement

The study was carried out in strict accordance with the recommendations described in the Guide for the Care and Use of Laboratory Animals of the National Institute of Health, the Office of Animal Welfare and the United States Department of Agriculture. All animal work was approved by the Oregon National Primate Research Center Institutional Animal Care and Use Committee (IACUC protocols # 0830 for mice and 0845 for NHP). The ONPRC has been continuously accredited by the American Association for Accreditation of Laboratory Animal Care since 1974 (PHS/OLAW Animal Welfare Assurance # A3304-01). The NHP work was conducted in BSL-3 containment where the environment was controlled for humidity, temperature and light (12 hour light/dark cycles). The NHP were housed in individual primate cages and fed twice a day with a standard commercial primate chow with water available ad libitum. Animals were monitored 4 times a day after infection and IACUC approved score parameters were used to determine when animals should be euthenized. All NHP procedures were carried out under Ketamine anesthesia in the presence of veterinary staff and all efforts were made to minimize animal suffering. Mice were housed in ONPRC's vivarium in barrier filter covered cages with food and water supplied ad libitum. Mice were euthanized using Isoflurane and all efforts were made to minimize animal suffering.

### Cells and viruses

*Aedes albopictus* cells (C6/36, ATCC:CRL-1660) were grown in minimum essential medium Eagle (MEM; ATCC) supplemented with 10% Fetal bovine serum (FBS; HyClone Laboratories) and antibiotics at 28°C. African green monkey kidney cells (WHO-VERO; ATCC) cells were grown in Dulbecco's modification of Eagle's medium (DMEM; Cellgro) containing 10% FBS, 2 mM L-Glutamine, and antibiotics at 37°C. Yellow Fever Virus-17D (YFV-17D, WHO Yellow Fever Seed, lot 213-77) was obtained from the National Institute for Biological Standards and Control, Hertfordshire, UK. YFV-DakH 1279 (YFV-Dakar) and YFV-FNV (French neurotropic vaccine) were obtained from the World Reference Center for Emerging Viruses and Arboviruses after approval from Dr. Robert Tesh (University of Texas Medical Branch, Galveston, TX).

### Animals

BALB/c ByJ mice were purchased from the Jackson Laboratory and used at 7–12 weeks of age.

Eight female rhesus macaques (8–14 years of age) and 1 male rhesus macaque (5 years of age) were inoculated subcutaneously with 20 infectious units (ifu) to 4×10^5^ ifu of YFV-Dakar (1–3 animals/dose). Serum was collected 4–6 days after infection and all animals succumbed to infection.

### YFV-specific polyclonal antibody (PAb) and monoclonal antibody (MAb) reagents

YFV-specific hyperimmune mouse serum was pooled from 19 mice that had been vaccinated with H_2_O_2_-inactivated YFV-17D and boosted with the same vaccine or vaccinated and later infected with either live YFV-17D or YFV-FNV. For development of the 3A8.B6 YFV-specific hybridoma cell line, a mouse was immunized subcutaneously with 10 µg of H_2_O_2_-inactivated YFV-17D adsorbed to 0.05% aluminum hydroxide gel (Sigma-Aldrich), boosted with the same vaccine one month later, and then challenged intravenously with 3×10^5^ Plaque Forming Units (PFU) of French neurotropic virus (FNV) one month after booster vaccination. Two months post-challenge, the mouse was boosted intravenously with 10 µg of H_2_O_2_-YFV and splenocytes were fused to P3X63-Ag8.653 myeloma cells [13] (a gift from M.S. Diamond,) four days later as previously described. Hybridoma clones were screened for YFV-specificity through a previously developed ELISA [14]. Both polyclonal and monoclonal antibodies were purified by Affi-gel protein A media (Bio-Rad, Hercules, CA) and biotinylated using the EZ-Link Microsulfo-NHS-LC Biotinylation kit (Thermo Fisher Scientific) per the manufacturers' instructions.

### Plaque assay

Plaque assays were performed by incubating 0.2 mL aliquots of 10-fold serially diluted virus on Vero cells (50–80% confluent) in 6-well plates. After 1 h at 37°C, the wells were overlaid with 3 ml of 0.5% agarose in MEM containing 2.5% FBS, 2 mM glutamine and antibiotics and incubated for 4–10 days, depending on the strain of virus. Cell monolayers were fixed with 75% methanol/25% acetic acid, the agarose was removed, and the plaques were visualized by staining with 0.1% crystal violet in Phosphate Buffered Saline (PBS) containing 0.2% formaldehyde. The plates were washed with tap water and allowed to dry before counting plaques.

### Focus forming assay (FFA)

The FFA was performed by incubating 100 µl of 10-fold serially diluted virus with Vero cells (50–80% confluent) in 24-well plates. After 1 h at 37°C, the wells were overlaid with 0.5 ml 1% methylcellulose in DMEM containing 1% FBS. After 3–10 days (3 days for YFV-17D and 3 to 10 days for YFV-Dakar) of incubation, the methylcellulose overlay was removed and the wells were washed with PBS, followed by fixation with 1% paraformaldehyde in PBS. The cells were permeabilized and stained intracellularly with YFV-specific mouse monoclonal antibody (clone 3A8.B6, available upon request) for 3–4 hours followed by incubation for 1–2 hours with horseradish peroxidase (HPR)- conjugated goat-anti-mouse IgG (H+L) (Invitrogen, G21040). The focus-forming spots were visualized using ImmPACT VIP Peroxidase Substrate (Vector laboratories, SK-4605) according to manufacturer's instructions. Photographs were taken on the same day as staining.

### Tissue culture limiting dilution assay (TC-LDA)

The TC-LDA was performed by incubating 2×10^4^ C6/36 cells per well in flat bottom 96-well plates (Costar, cat. no. 3595) at 28°C in MEM containing 10% FBS, and antibiotics with serial dilutions of YFV (10 wells/dilution). Each plate also included 4–8 wells of uninfected cells as negative controls. After 1 to 10 days of incubation, the cells were resuspended using a multichannel pipette and approximately 200 µl were transferred to round bottom 96-well plates (BD, cat. no. 353077) containing 100 µl of 6% formaldehyde in PBS (final concentration, 2% formaldehyde). It is important to add the cells under the surface and mix to ensure that all virus comes into direct contact with the formaldehyde solution for consistent inactivation. Round bottom plates are used for the staining procedure to minimize cell loss during the washing steps. The cells are permeabilized, by washing 2 times with PermWash (0.1% saponin and 1% FBS in PBS), incubated with mouse IgG to block non-specific binding and then stained intracellularly with an optimized concentration of biotinylated YFV-specific monoclonal antibody (3A8.B6) for 1 hour at 4°C. The cells were washed 3 times with PermWash before incubation with Strep-Avidin-PE (InVitrogen, S866) for 30 minutes at 4°C. After washing the cells 3 times with PermWash and 2 times with FACS-buffer (1% FBS in PBS) they were resuspended in 70 µl of 2% formalehyde in PBS and acquired on a FACSCalibur (Becton Dickinson) using CellQuest software (Becton Dickinson). Analysis was performed using FlowJo software (TreeStar). Live cell gating was performed using forward and side scatter characteristics. The gate for determining YFV-positive versus YFV-negative events at 7 to 10 days post-infection was set so that the non-specific background staining of uninfected cells was approximately 0.02% (range, 0.01–0.06%). Individual wells were determined to be YFV-positive if the number of YFV-positive events were more than 4-fold higher than the average background. The TC-LDA titer was defined as the reciprocal of the virus dilution required for 37% of the wells to be negative (i.e., uninfected). Log-log transformation of the data was used to calculate the TC-LDA titer and then converted to the final values using at least 3 data points and with at least one data point on each side of 37% cutoff. Dilutions that were 100% negative were avoided for use in the calculations unless it was the only point above 37% negative. Dilutions that were 0% negative cannot be used for log-log calculations but 0.1% may be substituted for 0% if there were no other data points below 37% negative or if another data point was needed to reach the minimum of 3 data points for calculating virus titer of a given sample.

### RNA isolation

RNA isolation was performed using the ZR Viral RNA kit per the manufacturer's instructions (Zymo Research). Briefly, frozen supernatants from TC-LDA or rhesus macaque serum samples were thawed on ice, and 10 µL of each sample was transferred to a tube containing 30 µL of ZR Viral RNA Buffer. This mixture was bound to a Zymo-Spin IC Column by centrifugation at 16,000× *g* for 2 minutes. The flow-through was discarded, and the column was washed twice with 300 µL of RNA Wash Buffer. Residual wash buffer was removed by centrifugation, and the purified RNA was eluted with 12 µL of RNase-free water.

### Reverse transcription and PCR

Purified RNA was reverse transcribed using the High Capacity cDNA Reverse Transcription Kit (Applied Biosystems) following the manufacturer's instructions for 20 µL reactions. Briefly, 10 µL of each purified RNA or positive control RNA (extracted from serum obtained from a YFV-Dakar infected rhesus macaque) was reverse transcribed. RNase-free water served as a negative control. PCR was performed in 25 µL reactions using 2 µL of each cDNA synthesis reaction as template, 17.25 µL of 0.22 µM filter sterilized double-distilled water, 2.5 µL 10× Maxima Hot Start Taq Buffer (200 mM Tris-HCl, pH 8.3, 200 mM KCl, 50 mM (NH_4_)_2_SO_4_, Fermentas Life Sciences), 1.5 µL of 25 mM MgCl_2_ (Fermentas Life Sciences), 0.25 µL Maxima Hot Start Taq DNA Polymerase (Fermentas Life Sciences), 0.5 µL of a 10 mM dNTP mix (dATP, dCTP, dGTP, dTTP each at 10 mM, Promega Corporation), and 0.5 µL each of 10 µM YF P1 and 10 µM YF P2 [15] (P1: 5′ TAC CCT GGA GCA AGA CAA GT 3′, P2: 5′ GCT TTT CCA TAC CCA ATG AA 3′). These primers were chosen because they were specific to YFV and bound within a conserved region within the envelope. Reactions were mixed in 0.2 mL thin-walled PCR tubes, and thermal cycling was performed in an Applied Biosystems thermal cycler. Cycling conditions for the PCR were as follows: one cycle of 95°C for 4 minutes; 40 cycles of 95°C for 30 seconds, 55°C for 60 seconds, and 72°C for 30 seconds; one cycle of 72°C for 10 minutes. PCR products (463 bp) were visualized by electrophoresis in an ethidium bromide-4% Nusieve agarose (Lonza) gel in TBE buffer (100 mM Tris-HCl, ph 7.8, 100 mM boric acid, 2 mM EDTA). A 20 µL aliquot from each PCR reaction was mixed with 4 µL of 6× Orange DNA Loading Dye (Fermentas Life Sciences), and all 24 µL were loaded into the gel. O'GeneRuler 100 bp DNA ladder (Fermentas Life Sciences) was included in each row of wells.

YFV genome copy numbers were also measured by quantitative RT-PCR (qRT-PCR) using the following forward (5′CAC GGA TGT GAC AGA CTG AAG A 3′) and reverse (5′CCA GGC CGA ACC TGT CAT 3′) primers and probe (5′ 6-FAM- CGACTGTGTGGTCCGGCCCATC 3′BHQ). Standard curve was established using the following amplicon as template (CGA CTG TGT GGT CCG GCC CAT CCA CGG ATG TGA CAG ACT GAA GAG GAT GGC GGT GAG TGG AGA CGA CTG TGT GGT CCG GCC CAT CGA TGA CAG GTT CGG CCT GG). In these experiments, cDNA was subjected to 10 min at 95°C followed by 40 cycles of [15 sec at 95°C/1 min at 60°C]. Experiments were carried out using a StepOnePlus Real-Time PCR system from Applied Biosystems.

### Statistical analysis

Virus titers were calculated by the logarithmic plot of the percent of negative cultures versus the dilution of inoculum, as previously described [16]–[19] and quantitation of infectious virus was calculated as the reciprocal of the sample dilution at which 37% of the cultures were negative for virus-infected cells as determined by flow cytometry. An electronic copy of an Excel sheet used to calculate virus titers by limiting dilution analysis is available upon request. Linear regression was used to determine the correlation, with associated p-value, between YFV viremia titers measured by TC-LDA and qRT-PCR, using the Regression Data Analysis Tool in Excel (Microsoft Corporation).

## Results

### Focus forming assays detect YFV-17D but not YFV-Dakar

**Figure 1:**
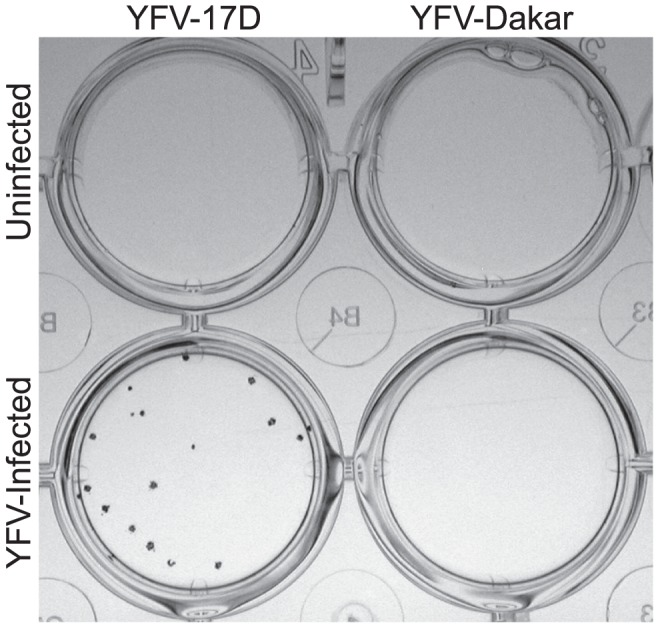
Quantitation of yellow fever virus by focus forming assay (FFA). This representative photograph shows the results of a FFA performed using uninfected Vero cells (top row) or Vero cells infected with YFV-17D (bottom left) or YFV-Dakar (bottom right). At 3 days post-infection, the monolayers were fixed and stained with anti-YFV Mab, 3A8.B6, and infectious foci were identified by addition of detection reagents as described in the methods. No focus forming units (FFU) could be detected in the YFV-Dakar-infected wells at 3, 7, or 10 days after infection with >100 infectious units of virus as determined by TC-LDA. The data is based on 4 experiments, each with similar results.

The vaccine strain of yellow fever, YFV-17D, is commonly used for *in vitro* studies and readily forms plaques on Vero cell monolayers. In contrast, after several attempts to optimize a plaque assay for quantitation of a low-passage stock of the clinical isolate, YFV-DakH1279 (YFV-Dakar), we realized that it did not form plaques or induce readily discernable cytopathic effect (CPE) in Vero cells or C6/36 cells. As an alternative approach, we developed a focus-forming assay (**Figure 1**) in which we fix virus-infected Vero cell monolayers and stain with YFV-specific monoclonal antibody, 3A8.B6, to identify infectious foci. This technique revealed focus-forming units (FFU) for YFV-17D after as little as 3 days of *in vitro* culture, but was not successful in detecting YFV-Dakar FFU even after extended incubation times of 3, 7, or 10 days in culture (**Figure 1** and data not shown).

### Development of a tissue culture limiting dilution assay for measuring infectious YFV

**Figure 2:**
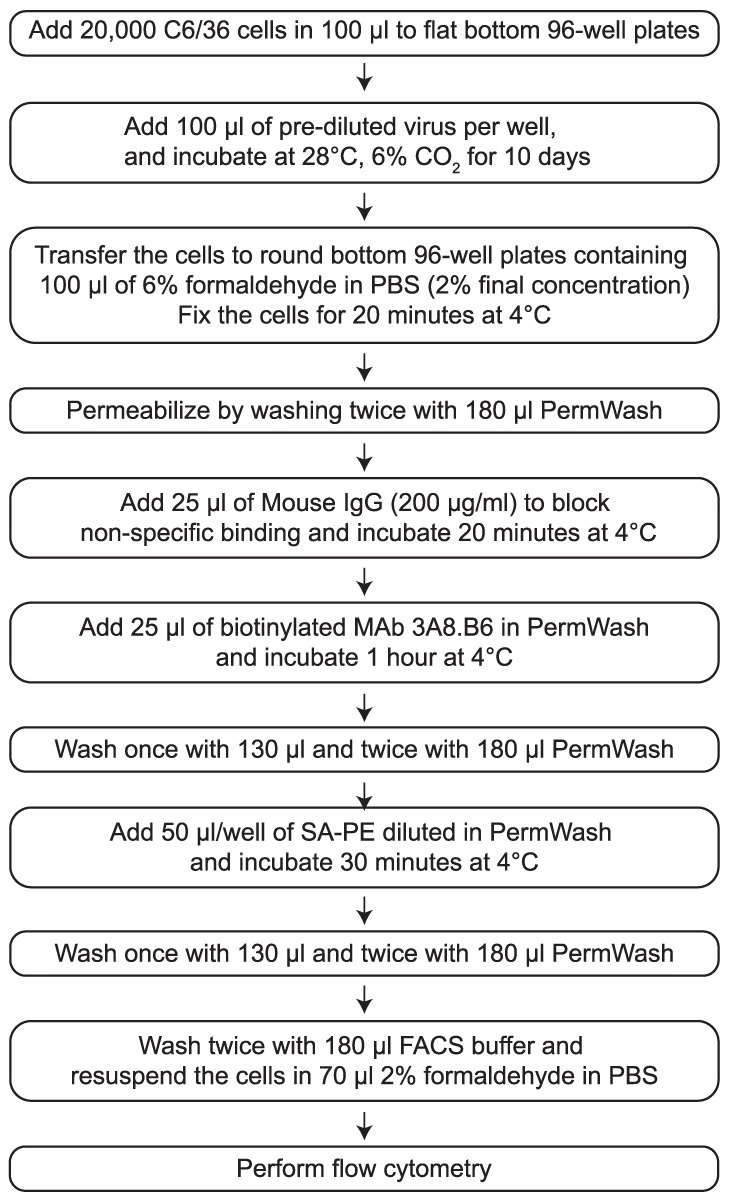
Outline of YFV tissue culture LDA (TC-LDA) methodology. For each washing step, the plates are centrifuged at 250× g for 3 minutes after which the solution is rapidly decanted into a waste container and the cell pellets are dislodged and resuspended by gentle vortexing before adding the next solution/reagent. Dilution of the biotinylated MAb 3A8.B6 and Strep-Avidin-R-phycoerythrin (SA-PE) were optimized for each lot. For instance, in the studies shown here, the biotinylated MAb 3A8.B6 was used at 8 µg/ml (4 µg/ml, final) and SA-PE was used at a final dilution of 1∶500.

**Figure 3:**
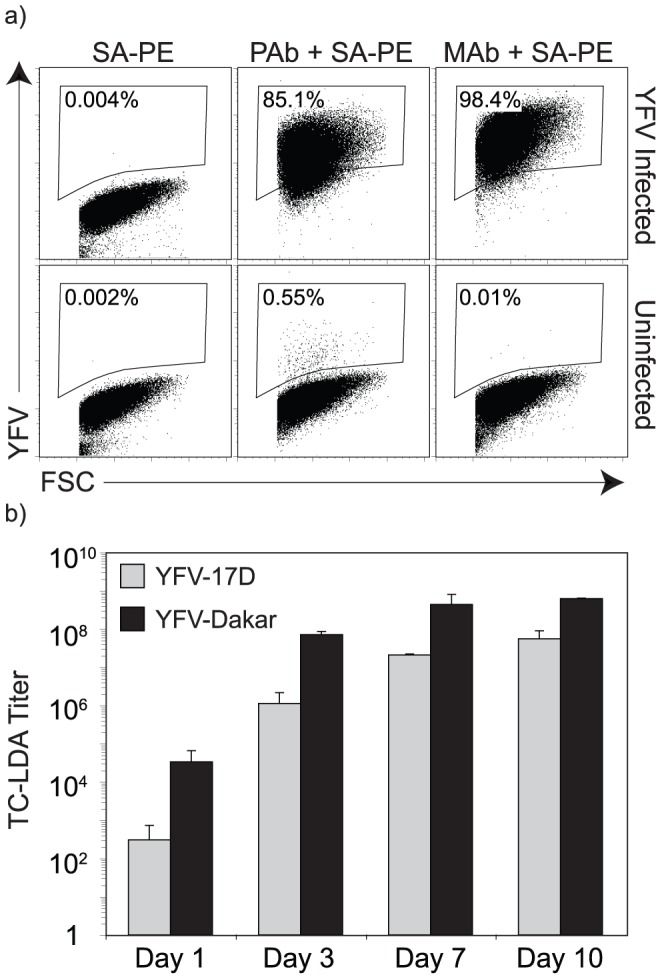
Optimization of YFV TC-LDA. In (A), representative flow cytometry dot plots show the results of staining uninfected C6/36 cells or YFV-17D-infected C6/36 cells (MOI = 5, 3 days post-infection) with biotinylated YFV-specific antibodies. Background staining with the secondary reagent, streptavidin-PE (SA-PE) in the absence of a primary YFV-specific antibody was low (<0.004%). Up to 85% of YFV-infected cells could be detected with biotinylated YFV-hyperimmune polyclonal mouse IgG (PAb + SA-PE) but about 98% of YFV-infected C6/36 cells could be detected with biotinylated MAb 3A8.B6 (MAb + SA-PE). In (B), the titer of two strains of YFV (YFV-17D and YFV-Dakar) were determined by TC-LDA (using MAb 3A8.B6) at 1, 3, 7, or 10 days after infection to determine the optimal incubation time required for sensitive quantitation of replicating virus. The data in (B) is based on the average of two independent experiments.

Fluorescence-based detection of flavivirus-infected cells by microscopy [20] or flow cytometry [21]–[23] has been used in the past to detect cells infected with flaviviruses and we reasoned that a similar approach could be optimized to identify YFV-Dakar by flow cytometry (**Figure 2**). As a proof-of-principle, C6/36 cells were infected with a high MOI of YFV-17D (MOI = 5) for 3 days and then fixed, permeabilized, and stained with biotinylated polyclonal YFV-specific hyperimmune antibody (PAb) or with biotinylated monoclonal antibody (MAb), 3A8.B6, followed by addition of streptavidin-PE (SA-PE) (**Figure 3**). Addition of the secondary SA-PE staining reagent alone showed very low background staining on uninfected or YFV-infected C6/36 cells. Use of polyclonal YFV-specific antibody was effective at identifying YFV-infected cells but the MAb, 3A8.B6, worked the best at detecting the highest frequency of infected cells (∼98%) with the lowest background binding to uninfected cells (0.01%) (**Figure 3A**).

Further optimization of the tissue culture limiting dilution assay (TC-LDA) began with determining the duration of *in vitro* tissue culture necessary for sensitive and accurate detection of YFV-infected cells (**Figure 3B**). Limiting dilution assays have been used previously to measure the frequency of memory T cells [24], [25] and memory B cells [6], [7] within a given population. In these current studies, the frequency of infectious virus particles per mL of sample was calculated by plotting the percentage of negative cultures versus the dilution factor and the virus concentration calculated as the reciprocal of the dilution at which 37% of the cultures are negative. This statistical approach is similar to the more common approach of defining a virus titer based on the reciprocal of the virus dilution in which 50% of cultures are negative [i.e., a tissue culture infectious dose 50 (TCID_50_)]. Virus titers based on the TCID50 methodology were approximately 1.27-fold higher than that determined by the statistically based TC-LDA, indicating that both approaches provide a similar estimation of infectious virus within a given sample. Using TC-LDA quantitation of infectious virus titers, we found that the estimated titer of two different strains of YFV (YFV-17D and YFV-Dakar) peaked by approximately 7 days of culture with no major improvement in assay sensitivity observed after 10 days of culture (**Figure 3B**). This indicates that infection of individual wells with infectious virus is maximally detectable by flow cytometry within 7–10 days of culture.

To determine the sensitivity and precision of the TC-LDA approach to virus quantitation, we measured the titers of two plaque-forming virus strains (YFV-17D and YFV-FNV) by plaque assay and TC-LDA (**Table 1**). We had previously determined that our optimized YFV plaque assay was 2–3-fold more sensitive than a focus-forming assay (data not shown) but were surprised to find that the TC-LDA was >10-fold more sensitive than the plaque assay for measuring infectious virus. Precision (i.e., reproducibility) was determined by calculating the inter-assay coefficient of variation (CV) and although there was some lot-to-lot variability, the precision was found to be similar for the plaque assay approach (17–51% CV) and the TC-LDA approach (7–31% CV) (**Table 1**). Together, this indicates that the TC-LDA is as reproducible as performing a plaque assay, but is more than 10-fold more sensitive at detecting infectious virus.

**Table 1:** Quantitation of infectious yellow fever virus by plaque assay and TC-LDA.

YFV Strain^a^	Plaque Assay Log_10_ PFU/mL (% CV)	TC-LDA Log_10_ ifu/mL (% CV)	Fold Difference
17D, P0	5.4 (51)	6.4 (7)	12
17D, P1	7.3 (30)	8.6 (9)	20
17D, P2	6.3 (30)	7.5 (31)	16
FNV, P1	7.0 (17)	8.5 (27)	34

a Plaque-forming yellow fever virus (YFV) strain YFV-17D-213-77 at Vero cell passage number 0, 1, 2 (P0, P1, and P2, respectively) and YFV-FNV (French neurotropic vaccine, Vero cell Passage 1) were quantitated by plaque assay or by flow cytometry-based tissue culture-limiting dilution assay (TC-LDA). The coefficient of variation (CV) indicates the inter-assay variability of the two independent approaches to virus quantitation based on 3–5 independent experiments. The variability (% CV) does not appear to be related to passage number since the YFV-FNV P1 stock has a similar TC-LDA CV to the YFV-17D P2 stock. YFV-Dakar was not included in these analyses since it did not form plaques on Vero cells (data not shown) and was not detectable by focus forming assay (Figure 1).

ifu; Infectious Unit.

**Figure 4:**
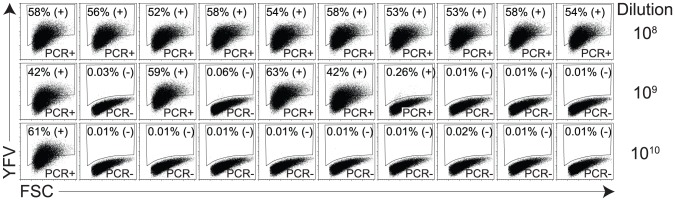
Independent analysis of YFV-infected cells by flow cytometry and RT-PCR. Dilutions of serum from a YFV-Dakar infected rhesus macaque (day 5 after infection with 1,000 infectious units of YFV-Dakar) were incubated with C6/36 cells (10 wells per dilution) for 10 days prior to removing 10% of the cells from each well for qRT-PCR analysis and then the remaining cells were fixed/stained for YFV antigen as described. The percentage of YFV-positive events shown in each dotplot represents the percentage of cells residing in the gated region that was set based on positive and negative controls included in the assay. Wells were considered positive by flow cytometry if the percentage of positive events were at least 4-fold over the background number of events observed in uninfected wells as indicated by (+) or (−). The titer of YFV-Dakar used in this experiment was calculated to be 4.1×10^9^ infectious units/mL and the sample dilutions performed in these representative dotplots (10^8^, 10^9^, 10^10^) is shown on the right-hand side of the figure. Wells that scored positive by YFV RT-PCR (PCR+) or negative by YFV RT-PCR (PCR-) are shown in the figure. There was 100% concordance between these two independent approaches to YFV detection.

As an independent measure of the accuracy of our flow cytometry-based approach to YFV detection, we performed a TC-LDA and verified the status of infected or uninfected culture wells by YFV-specific reverse transcriptase PCR (RT-PCR) (**Figure 4**). In this experiment, serum from a viremic YFV-Dakar-infected rhesus macaque was serially diluted, added to C6/36 cells (10 wells per dilution), and after 10 days of incubation, each well was tested for YFV-infected cells by flow cytometry with 10% of each well also tested for the presence of YFV by RT-PCR. We found 100% concordance between these two independent methods of virus detection; each well that scored positive for YFV antigen by flow cytometry (+) also scored positive for YFV RNA by RT-PCR and likewise, each well that scored negative by flow cytometry (−) also scored negative by RT-PCR.

### Use of TC-LDA approach to measuring infectious virus in YFV-Dakar-infected rhesus macaques

**Figure 5:**
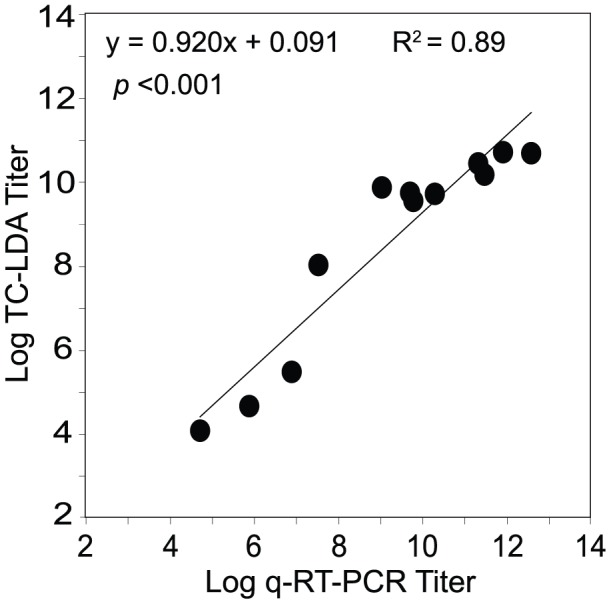
Correlation between serum YFV titers determined by TC-LDA and quantitative RT-PCR. The titer of YFV-Dakar in the serum of 12 infected rhesus macaques (4–6 days post-infection) were determined by TC-LDA, an approach that measures infectious virus, and by quantitative RT-PCR (qRT-PCR), an approach that measures the number of virus genome equivalents. The correlation (R^2^ = 0.89, *p*<0.0001) between these two independent and quantitative approaches indicates a nearly 1∶1 relationship between infectious virus and viral RNA genome equivalents when measuring *in vivo* infection by YFV-Dakar.

Analysis of viremia following infection with YFV represents an important reason for developing MAb 3A8.B6 and the YFV TC-LDA. YFV viremia is typically low following YFV-17D infection [12] and YFV RNA (i.e., RNAemia) if often more sensitive than plaque assay for monitoring virus replication *in vivo* [12]. To determine the relationship between RNAemia and viremia following experimental YFV-Dakar infection of non-human primates, we measured virus titers by quantitative RT-PCR (qRT-PCR) and TC-LDA, respectively (**Figure 5**). Serum samples were drawn between 4 and 6 days after infection with different doses of YFV-Dakar and the twelve serum samples tested in these experiments were chosen because they represented a greater than 10 million-fold range in virus titers based on qRT-PCR analysis. There was a strong and highly significant correlation between virus titers measured by TC-LDA and qRT-PCR (R^2^ = 0.89, *P*<0.0001; **Figure 5**) and the slope of the line indicates that there is approximately a 1∶1 relationship between the number of infectious virus particles and virus genome equivalents identified in these serum samples.

## Discussion

In these studies, we have generated a YFV-specific MAb, 3A8.B6 and used it to develop a flow cytometry-based assay for quantitation of low passage clinical isolates of yellow fever that do not develop appreciable CPE or form plaques *in vitro*. This approach is >10-fold more sensitive than a standard plaque assay for quantitating plaque-forming strains of yellow fever (e.g., YFV-17D and YFV-FNV) and can be used to reproducibly detect non-plaque-forming strains of yellow fever such as YFV-Dakar in tissue culture supernatants as well as from viremic serum samples from experimentally infected non-human primates.

RT-PCR is an effective tool for detecting YFV but it is based on detection of viral RNA and is not directly indicative of the levels of infectious virus. Indeed, in prior studies based on YFV-17D, the ratio of genome equivalents to infectious virus particles was found to range between 1000∶1 to 5,000∶1 [17]. This may have been due to studying YFV-17D harvested from cell culture supernatants or it may be due, at least in part, to the plaque assay approach being >10-fold less sensitive than the TC-LDA (Table 1). Interestingly, YFV RNAemia in human subjects infected with YFV-17D will reach >100 genome equivalents/mL [19] despite low infectious virus titers as measured by plaque assay that rarely reach 100 PFU/mL and typically reside below 20 PFU/mL ([12], [14] and data not shown). These results are in contrast to the data described here in which we found there is nearly a 1∶1 ratio between YFV-Dakar RNA genome equivalents and the levels of infectious virus as determined by TC-LDA (Figure 5). Again, these differences may be due to the lower sensitivity of plaque assay technology vs. TC-LDA measurements or this could reflect biological differences between YFV-17D and YFV-Dakar viremia/RNAemia. More studies will be needed to distinguish between these potential outcomes.

Although the use of a flow cytometry-based assay for virus quantitation requires optimization of staining procedures and flow cytometry gating procedures, it directly measures infectious virus (rather than genome-equivalents based on qRT-PCR) and provides some other useful advantages. For instance, viruses containing quasispecies can be cloned by terminal dilution in which cultures are theoretically infected with a single infectious virus particle. This would be accomplished by fixing/staining a portion of each culture well and once positive wells are identified, the remaining portion of the well contents can be utilized for downstream manipulations. By repeating this process until a homogeneous virus population is achieved, one could use this approach similar to plaque purification to select for genetically pure viruses with unique qualities and growth characteristics. Indeed, analysis of YFV-Dakar from the serum of an experimentally infected rhesus macaque (**Figure 4**) revealed individual wells containing viruses with different apparent growth characteristics. One well contained virus that infected just 0.26% of the C6/36 cells whereas other virus isolates infected 61–63% of the C6/36 cells when grown under identical conditions. Using this approach, it is possible to quantitate non-plaque forming strains of YFV, but this technique could also be used with other non-cytopathic viruses and provide a feasible approach to select/purify individual virus clones for further *in vitro* or *in vivo* study.
